# Antidiabetic Activity of *Clerodendron phlomoidis* Leaf Extract in Alloxan-Induced Diabetic Rats

**DOI:** 10.4103/0250-474X.49141

**Published:** 2008

**Authors:** S. P. Dhanabal, M. K. Mohan Marugaraja, B. Suresh

**Affiliations:** Department of Phytopharmacy and Phytomedicine (TIFAC CORE), JSS College of Pharmacy, Rocklands, Ootacamund-643 001, India

**Keywords:** Alloxan, antidiabetic activity, *Clerodendron phlomoidis*, ethanol extract

## Abstract

Ethanol extract of leaves of *Clerodendron phlomoidis* L. subjected to preliminary qualitative phytochemical investigations showed the presence of alkaloids, phytosterols, glycosides, saponins, phenolic compounds, proteins and flavonoids. The extract was screened for hypoglycemic activity in alloxan-induced diabetic rats (120 mg/kg, i.p.) at two dose levels, viz., 100 and 200 mg/kg. The ethanol extract at 200 mg/kg dose level exhibited significant (*p*<0.05) hypoglycemic activity and also correction of altered biochemical parameters viz., cholesterol and triglycerides (*p*<0.05).

Diabetes has been known to medical sciences longer than any other hereditary metabolic diseases. Nevertheless, the existing methods of treatment for this age old illness are not completely satisfactory. One area receiving particular attention today is that of herbal folk medicines. *Clerodendron phlomoidis* L. (Family: Verbenaceae) is commonly known as *Thazhu thaazhai* in Tamil and *Arni* in Hindi^1^,^2^. In traditional systems of medicine this species in useful in treating various diseases viz., juice of leaves is used as an alternative and bitter tonic. In southern India, the juice of the leaves is given in neglected syphilitic complaints in doses of half an ounce or more twice daily. The root decoction is slightly aromatic and astringent is used as a demulcent in gonorrhoea. It is also given to children during convalescence from measles. It is a bitter tonic, and is given in the convalescence of measles^1^,^2^. The leaves of this plant is being used as a remedy to treat diabetes in southern parts of India especially tribals of Nilgiris. But this species has not been scientifically evaluated for presence of active constituents and pharmacological activities. Hence, the present study is undertaken for its detailed study on presence of constituents and antidiabetic activity.

The leaves of *C. phlomoidis* were collected during December 2004 from Trichy, India. The plant species was identified and authenticated by comparing with the voucher specimen at the Survey of Medicinal Plants and Collection Unit, Government Arts College, Ootacamund, India. The leaves were dried under shade, crushed into coarse powder. The powder was defatted with petroleum ether (60-80°) and the defatted powder was loaded into Soxhlet extractor in 5 batches of 200 g each and was subjected to extraction for about 18–20 h with ethanol (95%). After extraction the solvent was distilled off and extract was concentrated on water bath to a dry residue and dried in a dessicator.

The ethanol extract was subjected to qualitative phytochemical investigation for the identification of the phytoconstituents viz., sterols, alkaloids, glycosides, saponins, tannins, carbohydrates and flavonoids^3^–^6^. Animal study protocols were approved by the Institutional Animal Ethics Committee. Healthy adult Wistar rats weighing 150-220 g were procured from the J. S. S College of Pharmacy animal house, Ootacamund, India. The animal house was well ventilated and animals had 12±1h day and night cycle. The animals were fed with rat pellet feed supplied by M/S Hindustan Lever Ltd., Bangalore, India and water *ad libitum*.

The rats were divided in to four groups of six animals each. Group I served as diabetic control and received 0.3% CMC, Group II served as positive control and received metformin (11.3 mg/kg), orally. Groups III and IV received the ethanol extract, orally at a dose of 100 mg/kg and 200 mg/kg respectively. The treatment was continued for seven days by administering the extract, drug or 0.3% CMC, once.

For the estimation of blood glucose, cholesterol (CHL) and triglyceride (TGL), the blood samples were collected by orbital sinus puncture under mild ether anesthesia in Eppendroff's tubes (1 ml) containing 50 μl of anticoagulant (10% trisodium citrate) and plasma was separated by centrifuging at 6000 rpm for 15 min and estimated in UV/Vis Spectrophotometer (Shimadzu). The absorbance of the sample and of the standard was measured against the reagent blank at 500 nm. The sample solution was prepared by adding 10 μl of the plasma and 1000 μl of the reagent blank, and the standard solution was prepared by adding 10 μl of the standard (glucose, CHL and TGL) with 1000 μl of the reagent blank. The values are expressed as mg/dl. Concentration of the sample = Absorbance of sample×concentration of standard/absorbance of standard

Experimental diabetes in overnight fasted Wistar rats was induced by a single intraperitoneal administration of alloxan monohydrate (120 mg/kg) in ice cold citrate buffer pH 4.5. Those animals with fasting blood glucose level between 170-300 mg/dl at 72 h after alloxan administration were divided into four groups of six animals each. Group I served as diabetic control and received 0.3% CMC, Group II served as positive control and received metformin (11.3 mg/kg), orally. Groups III and IV received the ethanol extract, orally at a dose of 100 mg/kg and 200 mg/kg. The treatment was continued for seven days by administering the extract, drug or 0.3% CMC, once daily. After the treatment period the blood glucose level, cholesterol (CHL) and triglyceride (TGL) were estimated. After the treatment period the rats were sacrificed and their pancreas were isolated for histopathological studies. The histological results were recorded on microphotographs. A small portion of each tissue was fixed in a 10% solution of formalin (formaldehyde) in 0.9% saline. These tissues were processed for paraffin embedding and sections were stained with haematoxylin-eosin (H and E) reagent and examined for intracellular changes.

The quantitative measurements in all the experiments were made on 6 animals in each group and the values are expressed as mean±SE. The data were subjected to one way ANOVA to determine the significance of changes followed by Dunnett's multiple comparisons to analyze the significance of difference within the experimental groups. *P* values of <0.05 were taken as significant.

The average percentage yield of ethanol (95%) extract of leaves of *C. phlomoidis* was found to be 3.92% w/w. The qualitative phytochemical tests carried out for the identification of the nature of phytoconstituents present in ethanol extract of leaves of *C. phlomoidis* showed mainly the presence of alkaloids, phytosterols, glycosides, saponins, phenolic compounds, proteins and flavonoids.

As expected in alloxan treated rats, there was significant increase in blood glucose, CHL and TGL levels. Oral treatment with 100 and 200 mg/kg of ethanol extract of *C. phlomoidis* significantly reduced the hyperglycemia. Maximum percentage of activity was shown by *C. phlomoidis* (200 mg/kg) -46.54% (*p*<0.05), while the standard metformin showed -50.55% (*p*<0.05, Table 1). In diabetic control group there was a significant increase in CHL and TGL levels. The standard metformin, and the ethanol extract of *C. phlomoidis* (100 and 200 mg/kg) used in the experimental study significantly (*p*<0.05) decreased the levels of CHL and TGL (Table 2).

**TABLE 1:** EFFECT OF ETHANOL EXTRACT ON PLASMA GLUCOSE LEVEL IN THE NORMAL, EUGLYCEMIC, DIABETIC AND TREATED RATS

Groups	Dose	Glucose level (mg/dl)				Activity (%)
			
		Normal	Euglycemic	After alloxan	After treatment	
Diabetic control (0.3% CMC)	-	63.21±2.88	69.97±1.85	179.90±10.95	209.5±16.580	+16.45
Positive Control (Metformin)	11.3 mg/kg	69.55±6.15	75.37±3.21	203.31±18.17	100.53±13.90*	−50.55
Ethanol extract of *Clerodendron phlomoidis*	100 mg/kg	77.15±3.04	77.80±2.79	217.52±14.17	154.40±16.46	−29.02
Ethanol extract of *Clerodendron phlomoidis*	200 mg/kg	80.86±3.38	72.91±5.86	210.68±26.76	112.61±29.05*	−46.54

Values are mean±SE, n=6. + denotes increase and - denotes decrease in hyperglycemic activity. The superscript

* denote statistical significance in comparison to after drug treatment respectively at *p* < 0.05.

**TABLE 2:** EFFECT OF ETHANOL EXTRACT ON CHOLESTEROL AND TRIGLYCERIDE IN NORMAL RATS AND AFTER TREATMENT

Group	Dose	Cholesterol level		Triglyceride level	
			
		Normal rats	After treatment in diabetic rats	Normal rats	After treatment in diabetic rats
Diabetic control (0.3% CMC)	-	52.05±3.01	168.70±19.06	72.81±15.29	160.62±16.17
Positive Control (Metformin)	11.3 mg/kg	61.54±6.81	81.67±1.77*	44.39±4.26	122.21±7.47*
Ethanol extract of *Clerodendron phlomoidis*	100 mg/kg	67.56±5.99	143.97±16.21	61.75±9.52	145.66±4.39
Ethanol extract of *Clerodendron phlomoidis*	200 mg/kg	76.28±5.13	120.05±6.50*	80.52±6.42	121.97±5.63*

Values are mean±SE, n=6. The superscript

* denote statistical significance in comparison to after drug treatment respectively at *p* < 0.05.

The purpose of choosing alloxan monohydrate as the diabetes-inducing agent was that it is known to produce diabetes mellitus irreversibly with a single dose administration by selective necrotic action on the beta cells of pancreas^7^ leading to insulin deficiency. Insulin deficiency leads to various metabolic aberrations in animals viz., increased blood glucose level^8^, decreased protein content^9^, increased levels of cholesterol and triglyceride^10^,^11^. It is well known that the level of glycemic control is the major determinant of serum level of triglyceride^12^. Several investigators demonstrated that near normalization of the blood glucose level resulted in significant reductions in levels of plasma cholesterol, and triglyceride level. Similar results were obtained with the seed powder of *Momordica charantia*^13^, *Momordica cymbalaria* fruit^14^ and jambolan fruit^15^. Similar antidiabetic activity was seen in the ethanol extract of leaves of *C. phlomoidis.*

Oral administration of ethanol extract of leaves of *C. phlomoidis* resulted in a significant reduction of serum lipid levels in rats with hyperlipidemia viz. triglyceride and total cholesterol. Flavonoids are known for their diverse biological activities including hypolipidemic activity. *C. phlomoidis* ethanol extract showed the presence of flavonoids and related phenolic compounds. Such dual property has also been reported in methanol extract of *Prunus dadidiana* (Rosaceae) and its flavonoid constituent, Prunin^16^.

Proteins and saponins have been reported to influence plasma cholesterol level^17^,^18^. As for proteins, the amino acid pattern of fenugreek seeds is similar to that of soya bean^19^. The hypocholesterolaemic effect of the ethanol extracts could possibly be related to its amino acid composition, since Chavsky^20^ showed that soya bean decreased cholesterol levels in rabbits and related this effect of amino acid pattern of this seed, particularly lysine/arginine ratio. The activities of two enzymes glucose-6-phosphatase and fructose-1,6-bisphosphatase in diabetic liver and kidney confirm fenugreek has insulin mimetic effect^21^.

In the histopathological studies more prominent islet cells were seen in the positive control and ethanol extract of *C. phlomoidis* (200 mg/kg) treated groups, while fairly prominent islets were seen in ethanol extract of *C. phlomoidis* (100 mg/kg) treated group and the diabetic control showed no endocrine glands. Oliver^22^ listed glycosides, flavonoids, tannins, alkaloids, coumarins, triterpenes, organic sulphur compounds and saponins as active hypoglycemic compounds. Hence the presence of reported constituents in the present study in the extracts may be responsible for hypoglycemic activity observed. Thus the plant species may possess both hypoglycemic and hypolipidemic activity as is known in traditional use.
